# The Implementation of Preconditioned Epidermal Neural Crest Stem Cells to Combat Ischemic Stroke. Comment on Othman, F.A.; Tan, S.C. Preconditioning Strategies to Enhance Neural Stem Cell-Based Therapy for Ischemic Stroke. *Brain Sci.* 2020, *10*, 893

**DOI:** 10.3390/brainsci11050653

**Published:** 2021-05-17

**Authors:** Sareh Pandamooz, Benjamin Jurek, Mohammad Saied Salehi, Mandana Mostaghel, Jaleel A. Miyan, Mehdi Dianatpour, Afshin Borhani-Haghighi

**Affiliations:** 1 Stem Cells Technology Research Center, Shiraz University of Medical Sciences, Shiraz 7193635899, Iran; mandana.mostaghel@gmail.com (M.M.); mdianatpur@gmail.com (M.D.); 2 Institute for Molecular and Cellular Anatomy, University of Regensburg, 93053 Regensburg, Germany; Benjamin.Jurek@vkl.uni-regensburg.de; 3 Clinical Neurology Research Center, Shiraz University of Medical Sciences, Shiraz 7193635899, Iran; saied_salehi@sums.ac.ir (M.S.S.); Neuro.ab@gmail.com (A.B.-H.); 4 Division of Neuroscience & Experimental Psychology, The University of Manchester, Manchester M13 9PT, UK; j.miyan@manchester.ac.uk

In the recent review published in *Brain Sciences*, Othman and Tan suggested several preconditioning strategies to improve stem cell therapy after ischemic brain injury [1]. They explored the regeneration efficacy of neural stem cells and introduced methods to optimize cell-based therapies. Due to some limitations related to the usage of neural stem cells, application of other pretreated cell types can also be beneficial for the ischemic brain [2]. Our research group has been studying the regenerative potential of epidermal neural crest stem cells (EPI-NCSCs) derived from hair follicles of rat and human since 2013 [3,4]. These multipotent stem cells are located in the bulge region of hair follicles throughout adulthood (Figure 1A), are ontologically related to the nervous system and present a high level of physiological plasticity [5]. Recently, we showed the therapeutic effect of transplanted EPI-NCSCs in a rat model of ischemic stroke, most likely through the simultaneous induction of neuronal and glial formation, as well as *Bdnf* overexpression. In this study, administration of EPI-NCSCs via intra-arterial or intravenous routes immediately after reperfusion, resulted in reduced infarct size seven days post transplantation (Figure 1B) [6]. Our previous findings also demonstrated that cerebrospinal fluid can be a practical route for the delivery of EPI-NCSCs [3]. Currently, our research group is focusing on the intranasal administration of EPI-NCSCs and their conditioned medium in the context of stroke. The preliminary data revealed that intranasal application of rat and human derived EPI-NCSCs and their secretome could alleviate the devastating condition following cerebral ischemia.

According to the work completed thus far, there are several preconditioning strategies to overcome poor cell survival, uncontrolled cell maturity, inefficient delivery and low engraftment rate, prior to the utilization of stem cells as viable clinical therapy. These preconditioning approaches, including hypoxic pretreatment, stem cell modification (neurotrophic overexpression), and chemical or biomaterial assistance, improve stem cell therapy and promote its clinical application.

The therapeutic benefit of hypoxic preconditioning applied to neural stem cells was also reviewed by Othman and Tan. Hypoxic pretreatment triggers various protective signaling pathways and increases cell survival and cytokine secretion. Interestingly, based on low oxygen tension in the hair follicles, ranging between 2.5% and 0.1% O_2_ [7], EPI-NCSCs are inherently resistant to low oxygen conditions. Thus, hypoxic preconditioning can mimic their physiological niche, which might improve their homing and neuroprotective ability following transplantation. Using chemical agents to optimize stem cell therapy is an approach where the evidence suggests that the application of various off-labelled drugs can improve the restorative potential of transplanted stem cells in animal models of ischemic stroke. Our previous experience supports this through the findings that small molecules, including valproic acid [8,9], dimethyl fumarate [10], and fingolimod [11] and acetylsalicylic acid (aspirin) can induce the expression of neurotrophic factors in EPI-NCSCs (Figure 1C).

Another preconditioning strategy involves the in vitro formation of three-dimensional (3D) stem cell aggregates that increase extracellular matrix secretion and promote their trophic functions. EPI-NCSCs can form spheres in vitro (Figure 1A) and transplantation of these spheres may present several advantages over conventional engraftment.

Othman and Tan also suggested applying an electrical current by means of conductive polymer to activate several intracellular signaling pathways in neural stem cells and promote their differentiation. Besides electrical stimulation to manipulate cell behaviors in vitro and in vivo, biomaterial assistance is an established preconditioning strategy to enhance stem cell function. Previously, we showed that providing a substrate with stiffness similar to the central nervous system elasticity enables EPI-NCSCs to retain more mechanical information about the future environment, which might improve their integration and enhance their function in the damaged neural tissue. This preconditioning resulted in overexpression of VEGF, NT3 and GDNF in these stem cells (Figure 1D) [12]. It is worth noting that EPI-NCSCs express several trophic factors such as BDNF, GDNF, NT3, NGF, and VEGF, all of which can benefit the inhospitable environment of the injured nervous system [13].

Despite the popularity of cell-based therapy, tracking the transplanted stem cells and evaluation of their therapeutic efficiency has been a major challenge in this field [14]. We previously reported the successful transduction of EPI-NCSCs with lentiviral particles encoding green fluorescent protein (Figure 1A), revealing some practical applications of this technique for indirect tracking of EPI-NCSCs [15].

Taken together, the review of Othman and Tan offered potential preconditioning strategies to improve neural stem cell transplantation. In parallel, we suggest EPI-NCSCs as a promising cell type for transplantation after cerebral ischemia. The implementation of the above-mentioned preconditioning modalities including hypoxic pretreatment, small molecules and biomaterial assistance or their combination along with using a proper route of administration can optimize the transplantation efficacy of EPI-NCSCs and their curative potential.

## Figures and Tables

**Figure 1:**
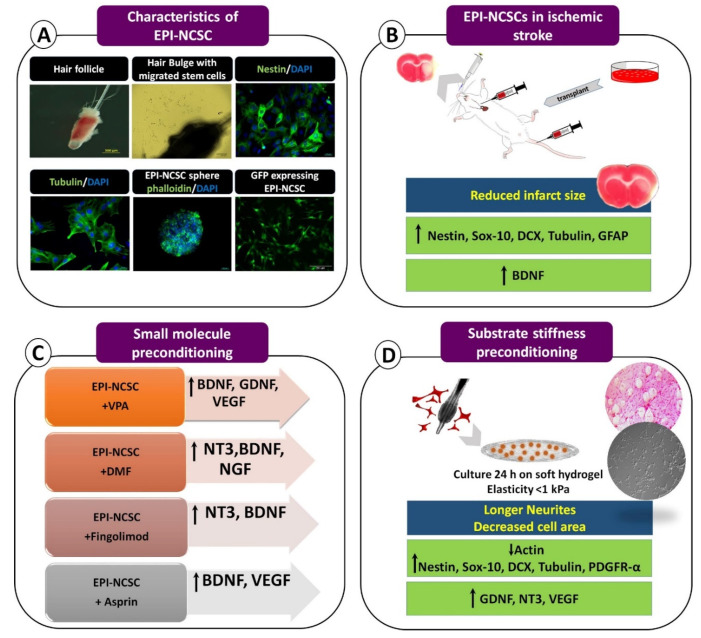
A summary of epidermal neural crest stem cells (EPI-NCSCs) characterization (**A**), benefit in a rat model of ischemic stroke (**B**), pretreatment with small molecules (**C**) and biomaterial assistance (**D**). DCX: doublecortin, GFAP: glial fibrillary acidic protein, BDNF: brain-derived neurotrophic factor, VPA: valproic acid, GDNF: glial cell-derived neurotrophic factor, VEGF: vascular endothelial growth factor, DMF: dimethyl fumarate, NT3: neurotrophin-3.
